# An Increase in Serum 25-Hydroxyvitamin D Concentrations Preceded a Plateau in Type 1 Diabetes Incidence in Finnish Children

**DOI:** 10.1210/jc.2014-1455

**Published:** 2014-11-01

**Authors:** Marjaana Mäkinen, Ville Simell, Juha Mykkänen, Jorma Ilonen, Riitta Veijola, Heikki Hyöty, Mikael Knip, Olli Simell, Jorma Toppari, Robert Hermann

**Affiliations:** 1 Department of Pediatrics, University of Turku, Turku, Finland and Turku University Hospital, Turku, Finland; 2 Immunogenetics Laboratory, University of Turku, Turku, Finland; 3 Department of Clinical Microbiology, University of Eastern Finland, Kuopio, Finland; 4 Department of Pediatrics, University of Oulu, Oulu, Finland and Oulu University Hospital, Oulu, Finland; 5 Department of Virology, University of Tampere, Tampere, Finland; 6 Fimlab Laboratories, Pirkanmaa Hospital District, Tampere, Finland; 7 Children’s Hospital, University of Helsinki and Helsinki University Central Hospital Helsinki, Finland; 8 Diabetes and Obesity Research Program, University of Helsinki, Helsinki, Finland; 9 Folkhälsan Research Center, Helsinki, Finland; 10 Department of Pediatrics, Tampere University Hospital, Tampere, Finland; 11 Department of Physiology, University of Turku, Turku, Finland

## Abstract

**Context:**

In Finland the world-record for the highest incidence of type 1 diabetes has risen steeply over the past decades. However, after 2006 the incidence rate has plateaued. We showed earlier, that despite the strong genetic disease component, environmental factors are driving the increasing disease incidence.

**Objective:**

Since vitamin D intake has increased considerably in the country since 2003, we analyzed how serum 25-hydroxyvitamin D (25[OH]D) concentration changed over time in healthy children, and the timely relation of these changes to disease incidence.

**Design, Setting and Participants:**

The birth cohort of the Finnish Type 1 Diabetes Prediction and Prevention project was used to explore longitudinal changes in serum 25-hydroxyvitamin concentrations. The sampling period was limited to children born from 1994 to 2004, with serum samples collected during 1998–2006 in the Turku area, Southwest Finland (60 °N).

**Main Outcome Measure:**

25(OH)D concentrations were measured every 3–6 months from birth, ages ranging from 0.3 to 12.2 years (387 subjects, 5334 measurements).

**Results:**

Serum 25(OH)D concentrations were markedly lower before 2003 than after (69.3 ± 1.0 nmol/L vs 84.9 ± 1.3 nmol/L, respectively, *P* < .001) in both genders. The mean difference between the periods was 15.7 ± 1.3 nmol/L (*P* < .001). Importantly, the frequency of children with low serum 25(OH)D levels (< 50 nmol/L) was reduced to almost half from 2003 (37.3% vs 69.9 %; *P* < .001). Similarly, severe vitamin D deficiency (<25 nmol/L) also decreased (2.7% vs 7.7%; *P* = .005). In addition, we detected higher 25(OH)D concentrations in young children (< 2 years) as compared to older children, which is explained by higher vitamin D intake in this group.

**Conclusions:**

We provide evidence that an increase in circulating concentrations of 25(OH)D shows a delayed temporal association with leveling off of type 1 diabetes incidence in Finland after 2006.

Finland has the highest incidence of type 1 diabetes (T1D) in the world. The mean age-specific incidence rate has increased constantly over the past decades from 32.4 per 100,000 person-years in 1980 to 64.9 per 100 000 person-years in 2006 (1), with similar trends described also in other populations (2). T1D is a complex disease, where the clinical phenotype usually develops as a result of a long disease process in genetically susceptible individuals. Environmental factors play a strong role in triggering and also probably in controlling the progression of β-cell autoimmunity. Identification of these environmental factors is of crucial importance, as they drive the rising disease incidence, and understanding them is likely to provide clues for disease prevention. We showed earlier, that an increasing environmental pressure and consequently higher penetrance of risk genes is likely responsible for this phenomenon, as the penetrance of low risk genotypes among new onset cases has increased during the last 50 years (3).

A recent observation by Harjutsalo et al showed that after 2006 the incidence rate of new childhood onset cases has plateaued in Finland (1). Vitamin D has been proposed as one of the environmental factors playing a role in the pathogenesis of T1D (4) and individuals with lower serum vitamin D levels have been proposed to have a higher risk for T1D (5). Accumulating data from epidemiological and clinical studies have indicated that vitamin D supplementation plays a role in maintaining a healthy immune system, thereby decreasing the risk for T1D. In Finland, vitamin D supplementation for infants and elderly has long been taken into account in healthcare policies, and in 2003 a new step was taken with the introduction of vitamin D fortification of milk products, which has been suggested as a possible reason for the change in the incidence rate (1).

We set out to test this hypothesis by analyzing serum 25-hydroxyvitamin D [25(OH)D] levels over a 9-year time period in a large prospective Finnish birth cohort.

## Materials and Methods

Subjects were healthy children participating in the Type 1 Diabetes Prediction and Prevention (DIPP) Study in Finland carrying HLA-conferred genetic risk for T1D (6). The DIPP project started in 1994 and it is an ongoing population-based prospective birth cohort study aimed at exploring the means to predict and prevent progression to clinical type 1 diabetes, as described previously (7). The first serum samples were collected at the age of 3 months and at 3- to 6-month intervals thereafter. The sampling period was limited to children born during the time period 1994–2004 with serum samples collected during 1998–2006 in the Turku area (latitude 60 °N) in order to avoid overrepresentation of young children and to encompass as large follow-up period as possible (7).

We measured 25(OH)D in 5334 serum samples from 387 children (233 boys, 154 girls, age range 0.3–12.2 years). The median number of samples per subject was 14 (range 1–37). The 25(OH)D analyses from these prospective serum samples were carried out during 2005–2013 using seven different batches of a commercial immunoassay kit (IDS Ltd.). Samples were stored at −75°C and all samples from each child were analyzed with the same batch of kits whenever possible. The results obtained in 2005–2007 with the first four kit batches were recalculated according to the instructions provided by the kit manufacturer and verified by the kit importer (Biofellows). The intra-assay coefficient of variation was 6.5% and the sensitivity reported by the kit manufacturer was 5 nmol/L. The performance target set by the Vitamin D External Quality Assessment Scheme Advisory Panel for 25(OH)D assays was met (8). The present study was conducted according to the guidelines of the Declaration of Helsinki, and was approved by the Ethics Committee of the Turku University Hospital. Written informed consent was obtained from all subjects and/or their guardians.

Statistical analyses were carried out using a mixed model method and χ^2^ tests. In the mixed model analyses the subject was used as a random effect. The effect of the dichotomized sample year was studied separately in two age groups (over/under 2 years) in order to reflect the different vitamin D recommendations of these groups (9). Dichotomized sample year (before/after 2003), sample month, and gender were used as independent variables. The associations were further studied by adding the interaction between the sample year and gender as independent variables. Statistical analyses were performed using SAS for Windows version 9.3. *P* values lower than .05 were considered statistically significant.

## Results

**Figure 1.**
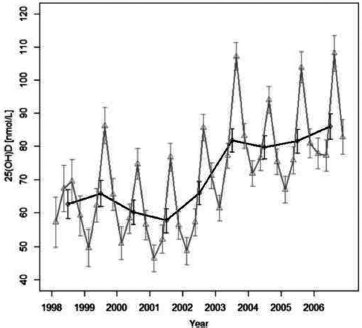
Variation of 25-hydroxyvitamin D levels in Finnish children older than 2 years of age. The black line indicates annual means, the gray line indicates quarterly means; error bars indicate 95% confidence interval (CI). (The plot is based on a model with sample month, sample year, and gender as predictors of vitamin D concentrations. The gray line is drawn from the model in which sample months have been replaced with yearly quarters and the interaction between quarters and sample year is added.)

Mean serum 25(OH)D concentrations were markedly lower during 1998–2002 than in the time period of 2003–2006 (69.3 ± 1.0 nmol/L vs 84.9 ± 1.3 nmol/L, respectively, *P* < .001) in both genders (Figure 1 and Table 1). Thus, the mean difference between the periods was 15.7 ± 1.3 nmol/L (*P* < .001). As expected, a clear seasonal variation was observed during the whole study period with peak values during late summer and the lowest values during late winter (Figure 1; *P* < .001). However, this did not influence the difference between the two time periods. In addition, we detected higher 25(OH)D levels in young children (< 2 years) as compared to older children (Table 1). Importantly, the frequency of children with low serum 25(OH)D levels (< 50 nmol/L) was almost halved from 1998–2002 to 2003–2006 (37.3% vs 69.9%; *P* < .001). Similarly, the frequency of severe vitamin D deficiency (<25 nmol/L) was significantly lower after 2002 than earlier (2.7% vs 7.7%; *P* = .005).

**Table 1.** 25-Hydroxyvitamin D Levels in Different Age Groups of Finnish Children, and Differences Between Time Periods of 1998–2002 and 2003–2006 (Means; 95% Confidence Interval)

Age group (y)	Gender (n)	25(OH)D (nmol/L) Years 1998–2002	25(OH)D (nmol/L) Years 2003–2006	Difference (nmol/L)	*P* Value
0–1.99	Boys (194)	78.0 (74.3–81.6)	88.7 (83.2–94.1)	10.7 (4.8–16.6)	.0006
0–1.99	Girls (120)	74.1 (69.8–78.4)	90.6 (78.7–102.4)	16.5 (4.0–29.0)	.0100
2.0–12.2	Boys (220)	64.6 (61.8–67.4)	80.3 (77.0–83.5)	18.7 (16.0–21.3)	<.0001
2.0–12.2	Girls (136)	60.0 (56.8–63.1)	83.3 (80.6–86.0)	20.3 (17.4–23.2)	<.0001

## Discussion

In this study, we provide evidence for the first time that increased 25(OH)D concentrations at the population level show a temporal association with a plateau in T1D incidence in Finland. Food items, mainly dairy products, have been supplemented with vitamin D (20 IU equivalent to 0.5 μg/100mL) since 2003 in Finland, which corresponds to at least 30–60 IU additional vitamin D intake in the age group of 0–6 years (9). Here we found, that this national vitamin D fortification policy clearly increased 25(OH)D serum concentrations in Finnish children. It is noteworthy, that prior to 2003 less than one-third of the children in our study had sufficient 25(OH)D levels (more than 50 nmol/L) and yet only 7.7% had very low levels (less than 25 nmol/L), leaving the majority in the range of 25–50 nmol/L. A systematic review of this field by Donnell et al (10) suggests that subjects with very low 25(OH)D levels benefit most from fortification of foodstuffs with vitamin D. In our study we showed that after the introduction of vitamin D supplementation in 2003, the prevalence of subjects with very low vitamin D (25(OH)D less than 25 nmol/L) was reduced to less than half. Also, the mean concentrations of 25(OH)D increased considerably, which supports the importance of food fortification during childhood in the general population. A constant marked increase in T1D incidence was observed in Finland between 1953 and 2004 (11), while the recommended daily vitamin D supplementation was reduced first from 4000 to 5000 IU to 2000 IU in 1964, and then to 1000 IU in 1975, and finally to 400 IU in 1992 (12). The recommended daily supplementation of vitamin D has been 300–400 IU in children ever since (13, 14). However, it is well known that the recommendations are often poorly followed so enzyme that the vitamin D intake remains lower than recommended (9) and food fortification might be a more efficient way to improve vitamin D intake than modifying the recommendations. It is noteworthy, that we detected higher 25(OH)D concentrations in young children (< 2 years) as compared to older children, which is probably explained by the higher compliance with the recommended vitamin D supplementation in the younger group (9).

The strengths of this study are several. First, it includes both sexes and a broad range of ages in a pediatric population. Second, the measurements were made in well-designed batches in a single laboratory thus minimizing variation (15). Third, to date, this study provides the largest number of serum 25(OH)D measured in consecutive follow-up samples, which permits for the first time the analysis of the seasonal changes in circulating 25(OH)D concentrations during different calendar years before and after vitamin D supplementation. The principal weaknesses of this study lie in the lack of data on vitamin D intake and supplement use. However, it seems unlikely that most children in this cohort would have abruptly increased their supplement use and vitamin D intake from other food sources simultaneously with the start of the vitamin D fortification. This was not seen in a very similar cohort receiving the same instructions on supplement use from the national health care system (9) or in another Finnish child cohort (16).

Our findings show that national vitamin D fortification can result in a significant increase in serum 25(OH)D concentrations in children. Similar findings have been obtained in a cohort of Finnish adults (17), but some have reported less significant effects in adults (18) than in children (16). All the other surveys have measured one serum sample before fortification and one sample after the fortification. As we show here, the seasonal variation in serum 25(OH)D concentrations is remarkable at northern latitudes, and relying on one or two samples is often not sufficient to determine the individual vitamin D status.

In conclusion, our findings show that the increased 25(OH)D concentrations observed since 2003 in Finnish children have a delayed temporal association with the reversal of the rising trend in the incidence T1D after 2006. This study does not answer the question whether vitamin D levels contribute to the pathogenesis of T1D, but the findings are in agreement with this hypothesis. It remains to be seen whether the incidences of other autoimmune diseases in children will be affected by the increase seen in circulating 25(OH)D concentrations.

## Abbreviations

CI: confidence interval
DIPP: Type 1 Diabetes Prediction and Prevention Project
25(OH)D: 25-hydroxyvatamin D
T1D: type 1 diabetes
